# Aloperine Suppresses the Tumorigenicity of Esophageal Squamous Cell Carcinoma by Targeting the AP-1/IL-6/STAT3 Signaling Axis

**DOI:** 10.3390/biom16060791

**Published:** 2026-05-27

**Authors:** Ba-Fang Ma, Jun-Nan Ye, Die Bai, Chang Ge, Yingchao Guan, Yang Lou, Ya-Ping Liang, Na Bu, Wenhui Hao, Yasen Maimaitiyiming

**Affiliations:** 1 Department of Immunology, School of Basic Medical Sciences, Xinjiang Medical University, Urumqi 830011, China; 107602230033@stu.xjmu.edu.cn (B.-F.M.); 107602230032@stu.xjmu.edu.cn (J.-N.Y.); 19992733269@stu.xjmu.edu.cn (D.B.); 1103093083@stu.xjmu.edu.cn (C.G.); 107602250030@stu.xjmu.edu.cn (Y.L.); 2 Xinjiang Key Laboratory of Molecular Biology for Endemic Diseases, Xinjiang Medical University, Urumqi 830011, China; 2095172052@stu.xjmu.edu.cn (Y.G.); lyp@stu.xjmu.edu.cn (Y.-P.L.); haowenhui@xjmu.edu.cn (W.H.); 3 Department of Biochemistry and Molecular Biology, School of Basic Medical Sciences, Xinjiang Medical University, Urumqi 830011, China; 4 Department of Pharmacy, Women’s Hospital, Zhejiang University School of Medicine, Hangzhou 310058, China; batiwangna@zju.edu.cn; 5 Institute of Basic Medical Sciences, School of Basic Medical Sciences, Xinjiang Medical University, Urumqi 830011, China

**Keywords:** Aloperine, esophageal squamous cell carcinoma, IL-6/STAT3 signaling, AP-1 transcription factor, apoptosis

## Abstract

Esophageal squamous cell carcinoma (ESCC) is an aggressive malignancy with a poor prognosis, largely due to therapeutic resistance and the limited availability of effective targeted therapies. Aloperine (ALO), a natural alkaloid derived from *Sophora alopecuroides* L., exhibits anti-cancer properties in various tumor types; however, its therapeutic potential and underlying mechanism in ESCC remain unclear. Here, we report that ALO inhibited ESCC cell proliferation and colony formation in a dose- and time-dependent manner and induced caspase-dependent apoptosis, accompanied by loss of mitochondrial membrane potential and PARP1 cleavage. Mechanistically, ALO significantly suppressed inflammatory pathways, with IL-6 identified as a critical downregulated target. ALO inhibited IL-6 production by targeting the AP-1 transcription factor complex, as evidenced by reduced cFOS expression and suppressed cJUN phosphorylation. Consequently, ALO inhibited downstream IL-6/JAK-STAT3 signaling. Functionally, exogenous IL-6 rescued ALO-induced loss of cell viability. Notably, the combination of ALO with cisplatin exerted synergistic antitumor effects. In a syngeneic mice model, the combination therapy significantly reduced tumor growth and Ki67 expression while inducing apoptosis, as shown by increased TUNEL staining and cleaved caspase-3 expression, and further suppressing the IL-6/STAT3 axis compared with either monotherapy. Together, these findings demonstrate that ALO exerts potent anti-ESCC activity by targeting the AP-1/IL-6/STAT3 signaling axis. The synergistic efficacy of ALO with cisplatin highlights its potential as a promising therapeutic agent to overcome chemoresistance and improve outcomes in ESCC patients.

## 1. Introduction

Esophageal squamous cell carcinoma (ESCC) represents the predominant histological subtype of esophageal cancer globally, accounting for nearly 90% of cases in high-incidence regions such as East Asia [1,2]. Despite advancements in multimodal therapies combining surgery, radiotherapy, and chemotherapy, the five-year survival rate for advanced ESCC remains poor, primarily due to late diagnosis, frequent metastasis, and the development of therapeutic resistance [3,4,5]. The pathogenesis of ESCC is a multistep process driven by the accumulation of genetic and epigenetic alterations [6], coupled with chronic inflammation triggered by risk factors such as tobacco use, alcohol consumption, and dietary carcinogens [7]. This inflammatory tumor microenvironment is a critical driver of ESCC progression, fostering proliferation, angiogenesis, and immune evasion [8]. Although significant progress has been made in delineating key oncogenic pathways [9], the intricate molecular network governing ESCC aggressiveness and therapy resistance remains incompletely understood, necessitating continued mechanistic exploration to identify novel therapeutic vulnerabilities [10].

The current standard first-line treatment for locally advanced ESCC includes neoadjuvant chemoradiotherapy followed by surgery, with platinum-based regimens (e.g., cisplatin) forming the therapeutic backbone [11,12]. For recurrent or metastatic disease, systemic chemotherapy, immunotherapy (e.g., PD-1 inhibitors), or their combination represents the mainstay of treatment, albeit with limited durable responses [12,13]. A major clinical challenge is the emergence of drug resistance, leading to therapeutic failure and disease relapse [14]. Both intrinsic and acquired resistance mechanisms, including enhanced DNA repair, activation of pro-survival signaling pathways, and alterations in the tumor microenvironment, contribute to this limitation [15]. Consequently, there is an urgent, unmet need to develop novel therapeutic agents, either as monotherapies or as sensitizers that can overcome resistance to existing modalities and improve clinical outcomes for ESCC patients [16].

Aloperine (ALO), a natural quinolizidine alkaloid isolated from the traditional herb *Sophora alopecuroides* L., has attracted increasing attention for its diverse pharmacological properties. It has been documented to exhibit broad bioactivities, including potent anti-inflammatory, anti-viral, anti-arrhythmic, and notably, anti-cancer effects across various malignancies [17]. Studies have reported that ALO can inhibit proliferation, induce apoptosis, and suppress metastasis in breast cancer [18], non-small-cell lung cancer [19,20], colorectal cancer [21,22], hepatocellular carcinoma [23], and prostate cancer [24], through modulating multiple pathways including PI3K/AKT, MAPK/ERK, and Ras. Its well-characterized anti-inflammatory activity, involving the suppression of key pathways like NF-κB [25], suggests a potential for modulating cytokine-driven oncogenic signaling, a hallmark of ESCC [26]. However, despite these promising activities, whether ALO possesses antitumor efficacy against ESCC and the precise molecular mechanisms underlying its potential action remain entirely unexplored.

In this study, we systematically investigated the anti-ESCC activity of ALO and delineated its underlying mechanism. Initial transcriptomic profiling of ALO-treated ESCC cells, combined with subsequent bioinformatic and functional analyses, revealed a profound suppression of the interleukin-6 (IL-6) signaling pathway. We demonstrated that ALO effectively inhibits IL-6 production primarily by targeting the AP-1 transcription factor complex, as evidenced by significant downregulation of phosphorylated cJUN (p-cJUN), concurrent reduction of cFOS expression, reduced binding of cJUN to the IL-6 promoter, and decreased AP-1 transcriptional activity. Given the established role of IL-6 in promoting ESCC progression [27], we further confirmed that ALO treatment potently reduces the phosphorylation and activation of STAT3 (p-STAT3). Importantly, we established that ALO synergizes with the standard chemotherapeutic agent cisplatin, both in vitro and in vivo, thereby significantly enhancing its cytotoxic efficacy. Our findings identify ALO as a promising therapeutic candidate for ESCC and provide a rationale for combining IL-6/STAT3 pathway inhibitors with conventional chemotherapy to overcome resistance and improve treatment outcomes.

## 2. Materials and Methods

### 2.1. Reagents

Aloperine, the pan-caspase inhibitor Q-VD-Oph, and cisplatin were purchased from MedChemExpress (MCE, Monmouth Junction, NJ, USA). SR11302 was purchased from Selleck Chemicals (Houston, TX, USA). Aloperine, Q-VD-Oph, and SR11302 were dissolved in dimethyl sulfoxide (DMSO) to prepare stock solutions. Cisplatin was dissolved in saline. All stock solutions were stored according to the manufacturers’ instructions. In all experiments, the final concentration of DMSO was kept at ≤ 0.1% for control and treatment groups. Recombinant human IL-6 protein (GMP-L06H27) was purchased from ACROBiosystems (Beijing, China). All other chemicals and reagents were of analytical grade.

### 2.2. Antibodies

The following primary antibodies were used in this study. Antibodies against GAPDH (60004-1-Ig), PARP1 (13371-1-AP), caspase-3 (82202-1-RR), cFOS (F0267), cJUN (24909-1-AP), STAT3 (60199-1-Ig), phospho-STAT3 (Ser727, 60479-1-Ig), Ki-67 (27309-1-AP), Bcl2 (68103-1-Ig), ERK1/2 (11257-1-AP), phospho-ERK1/2 (Thr202/Tyr204), phospho-JNK (Tyr185) (80024-1-RR), and Myc (67447-1-Ig) were purchased from Proteintech Group (Wuhan, China). The phospho-cJUN (Ser63) (AF5779) and JNK (AF1048) antibodies were purchased from Beyotime Biotechnology (Shanghai, China). The IL-6 antibody (F2097) was purchased from Selleck Chemicals (Houston, TX, USA). HRP-conjugated goat anti-rabbit IgG (SA00001-2) and HRP-conjugated goat anti-mouse IgG (A00001-1) secondary antibodies were also purchased from Proteintech Group.

### 2.3. Cell Lines and Culture Conditions

The human ESCC cell lines KYSE-150 and TE-1, the normal human esophageal epithelial cell line (HEEC), and the murine ESCC cell line AKR were obtained from Meisen CTCC (Zhejiang Meisen Cell Technology Co., Ltd., Hangzhou, China). KYSE-150, TE-1, and HEEC cells were cultured in RPMI-1640 medium (Gibco, Grand Island, NY, USA) supplemented with 10% fetal bovine serum (FBS; Gibco) and 1% penicillin–streptomycin (Gibco). AKR cells were cultured in DMEM (Gibco) supplemented with 10% FBS and 1% penicillin–streptomycin. All cells were maintained in a humidified incubator at 37 °C with 5% CO_2_.

### 2.4. Cell Viability Assay

Cell viability was assessed using the Cell Counting Kit-8 (CCK-8; Proteintech Group, Inc., Cat No. PF00004) according to the manufacturer’s protocol. Briefly, cells were seeded in 96-well plates at a density of 5 × 10^3^ cells per well and allowed to adhere overnight. Cells were then treated with various concentrations of ALO (0–1200 µM) or cisplatin for the indicated durations (24, 48, or 72 h). For combination studies, cells were treated with ALO and cisplatin either alone or in combination. The absorbance was measured at 450 nm using a microplate reader (Thermo Fisher Scientific, Waltham, MA, USA). The half-maximal inhibitory concentration (IC_50_) was calculated using GraphPad Prism 10 software.

### 2.5. Colony Formation Assay

For the colony formation assay, cells were seeded in 6-well plates at a density of 500–1000 cells per well. Following overnight attachment, cells were treated with the indicated concentrations of ALO. The medium was replaced every 3 days. After 10–14 days, colonies were fixed with 4% paraformaldehyde and stained with 0.5% crystal violet. Colonies containing more than 50 cells were counted under a microscope.

### 2.6. Apoptosis Analysis by Flow Cytometry

Apoptosis was quantified using the Annexin V-7AAD Apoptosis Detection Kit (MultiSciences Biotech Co., Ltd., Hangzhou, China)). Cells were seeded in 6-well plates and treated with ALO for 12 h. After treatment, cells were harvested, washed twice with cold phosphate-buffered saline (PBS), and resuspended in 1× binding buffer. The cells were then stained with Annexin V and 7-AAD for 15 min at room temperature in the dark. Apoptotic cells (early and late) were analyzed using a flow cytometer (BD FACSCanto II, San Jose, CA, USA) and FlowJo software (v10.8.2, FlowJo LLC, Ashland, OR, USA).

### 2.7. Live/Dead Cell Staining

To visualize cell viability and cytotoxicity, live/dead cell staining was performed using Calcein AM and 7-AAD. After ALO treatment, ESCC cells were washed twice with PBS and incubated with staining solution containing 2 μM Calcein AM and 4 μM 7-AAD (Beyotime Biotechnology, Shanghai, China) for 30 min at 37 °C in the dark. Calcein AM stains live cells with green fluorescence, while 7-AAD stains dead cells with red fluorescence. Images were captured using a laser scanning confocal microscope (Olympus, Tokyo, Japan). Merged images were analyzed to assess the proportion of live and dead cells.

### 2.8. Assessment of Mitochondrial Membrane Potential

Mitochondrial membrane potential (ΔΨm) was assessed using JC-1 dye (Beyotime Biotechnology, Shanghai, China). After ALO treatment, cells were harvested, washed with PBS, and incubated with JC-1 staining solution for 20 min at 37 °C. The cells were then washed twice with JC-1 buffer and analyzed using a flow cytometer. Carbonyl cyanide m-chlorophenyl hydrazone (CCCP) was used as a positive control. The ratio of red (JC-1 aggregates) to green (JC-1 monomers) fluorescence was calculated as an indicator of ΔΨm.

### 2.9. Western Blot Analysis

Cells or tumor tissues were lysed in RIPA buffer (Beyotime Biotechnology) supplemented with protease and phosphatase inhibitors (ServiceBio, Wuhan, China). Protein concentrations were determined using the BCA protein assay kit (Thermo Fisher Scientific, Waltham, MA, USA). Equal amounts of protein (20–30 μg) were separated by SDS-PAGE and transferred onto PVDF membranes (Millipore, Burlington, MA, USA). The membranes were blocked with 5% non-fat milk in TBST and then incubated with primary antibodies overnight at 4 °C. After washing, the membranes were incubated with HRP-conjugated secondary antibodies for 1 h at room temperature. Protein bands were visualized using enhanced chemiluminescence (ECL) reagents (Thermo Fisher Scientific) and imaged using an SCG-W2000 chemiluminescence imaging system (ServiceBio, Wuhan, China). Densitometric analysis was performed using ImageJ software (Version 1.52a, National Institutes of Health, Bethesda, MD, USA).

### 2.10. RNA Extraction and Quantitative Real-Time PCR (RT-qPCR)

Total RNA was extracted from treated cells using TRIzol reagent (Invitrogen, Carlsbad, CA, USA) according to the manufacturer’s instructions. RNA concentration and purity were assessed using a NanoDrop spectrophotometer (Thermo Fisher Scientific). Complementary DNA (cDNA) was synthesized from 1 μg of total RNA using a reverse transcription kit (Takara, Kyoto, Japan). RT-qPCR was performed using SYBR Green Master Mix (Takara) on a CFX96 Real-Time PCR Detection System (Bio-Rad Laboratories, Inc., Hercules, CA, USA). Relative gene expression was calculated using the 2^−ΔΔCt^ method, with GAPDH serving as an internal control. Primer sequences are shown in Table 1.

### 2.11. RNA Sequencing and Bioinformatic Analysis

TE-1 cells were treated with DMSO (control) or ALO for 6 h in triplicate (three biological replicates per group). Total RNA was extracted, and RNA-seq libraries were prepared and sequenced on a BGI sequencing platform (BGI, Shenzhen, China) by Honsun Bio (Shanghai, China). Differentially expressed genes (DEGs) were identified using the criteria |log_2_(fold change)| > 1 and adjusted *p*-value < 0.05. Gene Ontology (GO) and Kyoto Encyclopedia of Genes and Genomes (KEGG) enrichment analyses were performed to identify significantly enriched biological processes and pathways. Heatmaps and volcano plots were generated using the R programming language.

### 2.12. Enzyme-Linked Immunosorbent Assay (ELISA)

IL-6 protein levels in cell culture supernatants were measured using an IL-6 ELISA kit (NeoBioscience, Shenzhen, China) according to the manufacturer’s instructions. Briefly, after ALO treatment, cell supernatants were collected and centrifuged to remove debris. Samples were added to pre-coated plates, incubated, and then detected with biotinylated detection antibodies. The absorbance was measured at 450 nm using a microplate reader, and IL-6 concentrations were calculated using a standard curve.

### 2.13. Dual Luciferase Reporter Assay

TE-1 cells were co-transfected with an AP-1 reporter plasmid (pHBLuc-APIRE, Hanbio, Shanghai, China) containing AP-1 binding sites upstream of the Firefly luciferase reporter, together with the Renilla internal control plasmid pRL-TK (Miaoling Plasmid Platform, Wuhan, China), using Lipofectamine 3000. After 24 h, cells were seeded into white 96-well plates and treated for 12 h with DMSO, ALO, and SR11302. Firefly and Renilla luciferase activities were measured using the dual luciferase reporter gene assay kit (Yeasen, 11402ES60, Shanghai, China) on a PerkinElmer Multimode Plate Reader (VICTOR Nivo^TM^, PerkinElmer, Inc., Waltham, MA, USA). AP-1 activity was calculated as the Firefly/Renilla ratio. Three independent experiments were performed in triplicate.

### 2.14. Chromatin Immunoprecipitation (ChIP) Assay

TE-1 cells were treated with DMSO or ALO (600 μM) for 12 h, crosslinked with 1% formaldehyde, and quenched with glycine. Chromatin was sheared to 200–500 bp using an ultrasonic cell disruptor (XM-650DT, Xiaomei Ultrasonic Instruments, Kunshan, China). Immunoprecipitation was performed overnight at 4 °C with an anti-cJUN antibody (Proteintech, F0168) or normal rabbit IgG (negative control) using Protein A/G magnetic beads (Thermo Fisher Scientific, USA). After crosslink reversal, purified DNA was analyzed by qPCR using primers spanning the AP-1 binding site in the IL-6 promoter (forward: 5′-GCTGTGAGATGCCTGGAGAA-3′; reverse: 5′-GCATTGCCAAAGAGTGAGTG-3′). Enrichment was calculated as percentage of input. Experiments were performed in triplicate.

### 2.15. Animal Studies and Xenograft Model

All animal experiments were approved by the Institutional Animal Care and Use Committee of Xinjiang Medical University (protocol code IACUC-JT-20240228-68, 28 February 2024). Male C57BL/6 mice (4–6 weeks old) were obtained from the Laboratory Animal Center of Xinjiang Medical University. To establish the xenograft model, AKR cells (5 × 10^6^) were suspended in 100 µL of PBS and subcutaneously injected into the right flank of each mouse. When tumors reached an average volume of approximately 100 mm^3^, mice were randomly divided into four groups (*n* = 6 per group): (1) Vehicle control, (2) ALO alone (10 mg/kg, administered intraperitoneally every 2 days), (3) Cisplatin alone (1 mg/kg, administered intraperitoneally every 2 days), and (4) ALO + Cisplatin combination (both at the same doses and schedule). Tumor volumes were measured every 3 days using a caliper and calculated using the formula: volume = (length × width^2^)/2. Body weights were monitored throughout the treatment period. At the end of the experiment, mice were euthanized, and tumors were excised, weighed, and photographed. Tumor tissues were then processed for Western blotting, immunohistochemistry, and TUNEL staining.

### 2.16. Immunohistochemistry (IHC) and TUNEL Staining

Tumor tissues were fixed in 4% paraformaldehyde, embedded in paraffin, and sectioned at 4 μm thickness. For IHC, sections were deparaffinized, rehydrated, and subjected to antigen retrieval. After blocking with 5% bovine serum albumin (BSA), slides were incubated with primary antibodies against Ki67 (Proteintech) overnight at 4 °C, followed by incubation with HRP-conjugated secondary antibody. Staining was visualized using 3,3′-diaminobenzidine (DAB), and sections were counterstained with hematoxylin. For apoptosis detection, TUNEL staining was performed using the In Situ Cell Death Detection Kit (Roche) according to the manufacturer’s protocol. Images were captured using a light microscope (Olympus, Tokyo, Japan), and positive cells were quantified in five random fields per section using ImageJ software.

### 2.17. Hematoxylin and Eosin (H&E) Staining

For histopathological examination of major organs (heart, liver, spleen, lungs, and kidneys) and tumor tissues, specimens were fixed in 4% paraformaldehyde, embedded in paraffin, and sectioned at 4 μm thickness. Sections were deparaffinized in xylene, rehydrated through a graded ethanol series, and stained with hematoxylin for nuclear visualization followed by eosin for cytoplasmic staining. After dehydration and clearing, sections were mounted with neutral balsam. Stained sections were examined and imaged under a light microscope (Olympus) to assess tissue morphology and any treatment-related toxicity.

### 2.18. Statistical Analysis

All experiments were performed in triplicate unless otherwise specified. Data are presented as the mean ± standard deviation (SD) or standard error of the mean (SEM). Statistical analyses were performed using GraphPad Prism 8.0 software. Comparisons among multiple groups were performed using one-way or two-way ANOVA followed by Tukey’s or Bonferroni’s post hoc test. Statistical significance was defined as follows: * *p* < 0.05, ** *p* < 0.01, and # *p* < 0.001.

## 3. Results

### 3.1. Aloperine Suppresses Proliferation of ESCC Cells in a Dose- and Time-Dependent Manner

**Figure 1:**
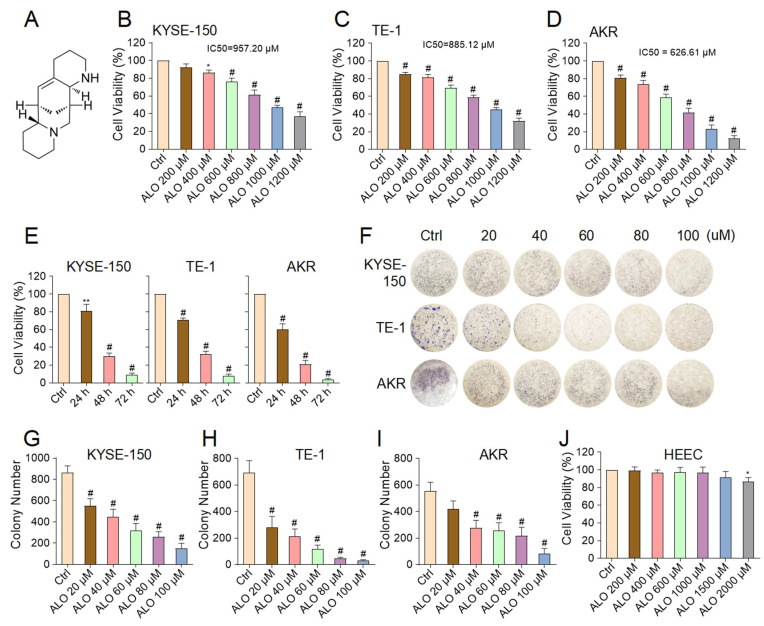
Aloperine inhibits ESCC cell proliferation. (**A**) Chemical structure of aloperine (ALO). (**B**–**D**) Dose-dependent inhibition of cell viability in human ESCC cell lines KYSE-150, TE-1, and murine ESCC AKR cells, as measured by CCK-8 assay after 24 h treatment. Data are presented as the mean ± SD, *n* = 3. (**E**) Time-dependent inhibition of cell viability in KYSE-150, TE-1, and AKR cells treated with 600 µM ALO. Data are presented as the mean ± SD, *n* = 3. (**F**) Representative images of colony formation in KYSE-150, TE-1, and AKR cells treated with 0–100 µM ALO for 10 days. (**G**–**I**) Quantification of colony formation in KYSE-150, TE-1, and AKR cells after 24 h treatment. (**J**) Dose dependent effects of ALO on HEEC cell viability. Data are presented as the mean ± SD, *n* = 3. * denotes *p* < 0.05, ** denotes *p* < 0.01, and # denotes *p* < 0.001 as compared to Ctrl (one-way ANOVA with Tukey’s post-test).

We first evaluated the anti-proliferative effects of ALO (Figure 1A) using the CCK-8 assay across a panel of ESCC cell lines. Cells were treated with ALO at concentrations ranging from 0 to 1200 µM for 24 h. As shown in Figure 1B–D, ALO induced a dose-dependent reduction in cell viability in all three ESCC lines. The calculated half-maximal inhibitory concentrations (IC_50_) were 957.2 µM (KYSE-150), 885.1 µM (TE-1), and 626.6 µM (AKR), indicating that the murine AKR cells were the most sensitive to ALO treatment. To further characterize the temporal dynamics of ALO’s effect, we treated the three ESCC cell lines with 600 µM ALO for up to 72 h. Cell viability decreased progressively over time in all cell lines, confirming that ALO’s growth-inhibitory effect is both concentration- and time-dependent (Figure 1E).

Concurrent with the viability data, morphological changes induced by ALO treatment were evident in all ESCC cell lines. As shown in Figure S1A–C, representative phase-contrast images of TE-1, KYSE-150, and AKR cells treated with increasing concentrations of ALO revealed progressive cell shrinkage, rounding, and detachment. These morphological alterations became more pronounced with higher doses and longer exposure times, visually corroborating the dose-dependent growth inhibition observed in the CCK-8 assays. To assess the long-term proliferative capacity of ESCC cells under ALO treatment, we performed colony formation assays. Low concentrations of ALO (20–100 µM) significantly reduced both the number and size of colonies in all three ESCC cell lines in a dose-dependent manner (Figure 1F–I), consistent with its ability to suppress sustained proliferation and clonogenic survival.

Finally, to evaluate the selectivity of ALO, we examined its effects on the normal human esophageal epithelial cell line HEEC (Figure 1J). Only at the highest concentration tested (2000 µM) did ALO exhibit minimal cytotoxicity toward HEEC, suggesting a favorable therapeutic window for targeting malignant ESCC cells while sparing normal epithelial counterparts. These results suggest that ALO selectively targets ESCC cell viability.

### 3.2. Aloperine Induces Apoptosis in ESCC Cells

**Figure 2:**
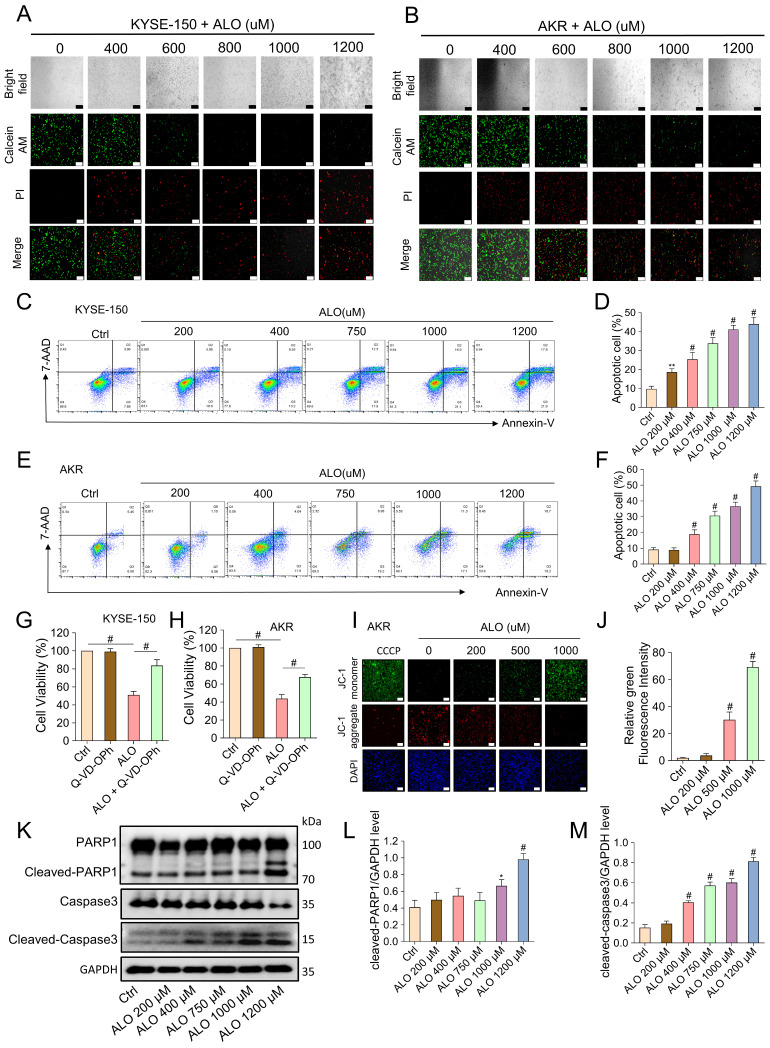
Aloperine induces apoptosis in ESCC Cells. (**A**,**B**) Live/dead cell staining of KYSE-150 (**A**) and AKR (**B**) cells treated with ALO (0–1200 µM) for 24 h. Bright field (**top**), calcein AM (live, green), 7-AAD (dead, red), and merged images are shown. Scale bar is 100 µm. (**C**–**F**) Annexin V-PI flow cytometry dot plots and quantification of apoptotic KYSE-150 (**C**,**D**) and AKR (**E**,**F**) cells treated with ALO (0–1200 µM) for 24 h. Data are presented as the mean ± SD, *n* = 3. (**G**,**H**) Viability changes of KYSE-150 and AKR cells co-treated with ALO (600 µM) and/or Q-VD-Oph (20 µM) for 24 h, as measured by CCK-8. Data are presented as the mean ± SD, *n* = 3. (**I**) JC-1 staining of AKR cells treated with ALO (0–1000 µM) for 24 h. JC-1 monomers are shown in green, JC-1 aggregates in red, and nuclei in blue. Scale bar is 100 µm. (**J**) Quantification of JC-1 green fluorescence intensity. Data are presented as the mean ± SD, *n* = 3. (**K**) Western blot analysis of PARP1, cleaved-PARP1, caspase-3, and cleaved-caspase-3 in KYSE-150 cells treated with ALO (0–1200 µM) for 24 h. GAPDH was used as a loading control. (**L**,**M**) Quantification of cleaved-PARP1 and cleaved-caspase-3 levels normalized to GAPDH. Data are presented as the mean ± SD, *n* = 3. * denotes *p* < 0.05, ** denotes *p* < 0.01, and # denotes *p* < 0.001 (one-way ANOVA with Tukey’s post-test). Original western blot images can be found in the Supplementary Materials.

To determine whether ALO-mediated growth inhibition involves apoptosis, we first performed live/dead cell staining in KYSE-150 and AKR cells. As shown in Figure 2A,B and Figure S2, bright-field images revealed reduced cell density with increasing ALO concentration, while calcein AM (green, live cells) fluorescence diminished and 7-AAD (red, dead cells) staining intensified, particularly at concentrations higher than 400 µM. Merged images confirmed a progressive shift from viable to non-viable populations.

We next quantified apoptosis via Annexin V-7AAD flow cytometry. In KYSE-150, early and late apoptotic cells increased significantly with ALO treatment, rising from ~10% in control group (Ctrl) to ~45% at 1200 µM for 12 h (Figure 2C,D). Similarly, AKR cells exhibited a dose-dependent increase in apoptosis, reaching approximately 50% at 1200 µM for 12 h (Figure 2E,F).

To confirm that apoptosis underlies ALO’s cytotoxic effect, we co-treated cells with the pan-caspase inhibitor Q-VD-Oph. In both KYSE-150 and AKR (Figure 2G,H), Q-VD-Oph significantly rescued cell viability loss induced by ALO, suggesting that caspase-dependent apoptosis contributes to ALO’s mechanism of action. We then assessed mitochondrial membrane potential (ΔΨm) using JC-1 dye. In control cells, prominent red fluorescence, representing JC-1 aggregates, indicated high ΔΨm. After ALO treatment, red fluorescence progressively decreased, whereas green fluorescence, representing JC-1 monomers, increased in a concentration-dependent manner, indicating mitochondrial depolarization (Figure 2I,J). CCCP was used as a positive control.

Western blot analysis further revealed cleavage of PARP1 and caspase-3 in both cell lines upon ALO treatment (Figure 2K). Further quantification confirmed a dose-dependent increase in cleaved PARP1 and cleaved caspase-3 levels (Figure 2L,M), further validating activation of the intrinsic apoptotic pathway. These results suggest that ALO induces apoptosis in ESCC cells.

### 3.3. Aloperine Inhibits IL-6 Production in ESCC Cells

**Figure 3:**
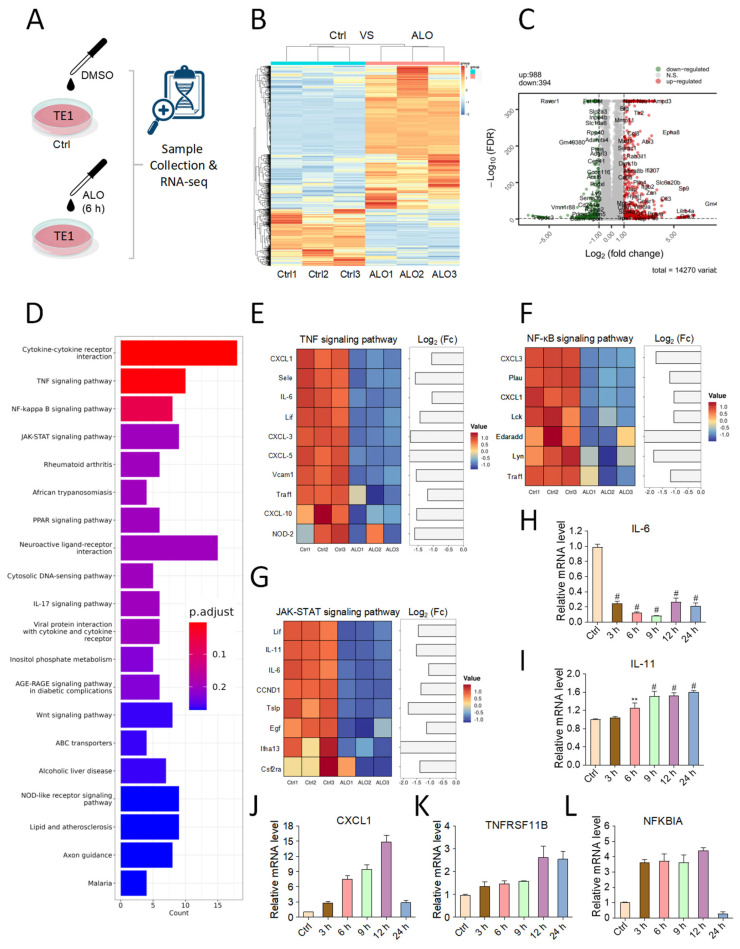
Aloperine inhibits IL-6 production in ESCC Cells. (**A**) Schematic of experimental design. TE1 cells were treated with DMSO (Ctrl) or ALO for 6 h before RNA sequencing. (**B**) Heatmap of differentially expressed genes (DEGs) between Ctrl and ALO-treated groups. *n* = 3 per group. (**C**) Volcano plot of differentially expressed genes. Red dots indicate significantly upregulated genes, green dots indicate downregulated genes, and gray dots indicate non-significant genes. (**D**) KEGG pathway enrichment analysis of downregulated genes after ALO treatment. (**E**–**G**) Heatmapsof representative genes in the TNF signaling, NF-κB signaling, and JAK-STAT signaling pathways. (**H**–**L**) RT-qPCR validation of key genes from the above pathways. Data are presented as the mean ± SD from three independent experiments. ** denotes *p* < 0.01, and # denotes *p* < 0.001 as compared to Ctrl (one-way ANOVA with Tukey’s post-test).

To elucidate the molecular mechanisms underlying ALO-mediated effects, we first treated TE-1 cells with DMSO (control) or ALO for 6 h and then performed RNA sequencing (Figure 3A). Comparative transcriptomic analysis revealed widespread changes in gene expression, as visualized by a heatmap and volcano plot (Figure 3B,C). In total, 988 genes were upregulated and 394 were downregulated after ALO treatment.

To gain a comprehensive view of the biological processes and signaling pathways affected by ALO, we performed Gene Ontology (GO) and KEGG enrichment analyses on all differentially expressed genes. GO analysis showed enrichment of processes related to ion homeostasis, immune regulation, and metabolism (Figure S3A). KEGG pathway analysis of all DEGs identified significant enrichment in pathways including neuroactive ligand–receptor interaction, cytokine–cytokine receptor interaction, the TNF signaling pathway, and steroid biosynthesis (Figure S3B).

To focus on pathways potentially contributing to ALO’s antitumor activity, we performed KEGG analysis specifically on the downregulated genes (Figure 3D). This analysis confirmed marked suppression of inflammation-associated pathways, among which cytokine–cytokine receptor interaction, the TNF signaling pathway, and the NF-κB signaling were the most prominently downregulated. Heatmaps of the top three suppressed pathways, TNF signaling (Figure 3E), NF-κB signaling (Figure 3F), and JAK-STAT signaling (Figure 3G), demonstrated consistent downregulation of key inflammatory and proliferative genes.

We next validated these transcriptomic findings by RT-qPCR analysis of selected genes from these pathways. Among the tested targets, only *IL-6* mRNA was significantly and consistently reduced at multiple time points after ALO treatment (Figure 3H). In contrast, expression of *IL-11*, *CXCL1*, *TNFRSF11B*, and *NFKBIA* showed no statistically significant changes (Figure 3I–L). These results suggest that ALO does not exert its primary effect through the canonical NF-κB signaling pathway, which is a major regulator of IL-6 transcription. Instead, ALO selectively and robustly inhibits *IL-6* transcription in ESCC cells, positioning IL-6 as a key downstream effector of the antitumor activity of ALO.

Notably, analysis of the Cancer Cell Line Encyclopedia (CCLE) database revealed that TE-1 and KYSE-150 exhibited the highest basal *IL-6* expression among ESCC cell lines (Figure S4). This finding supports the rationale for using these two high-IL-6-expressing cell lines as the primary human models in this study and provides a strong biological basis for investigating the effects of ALO on IL-6-driven oncogenic signaling in ESCC.

### 3.4. ALO Inhibits IL-6/JAK-STAT Signaling Pathway by Targeting AP-1

**Figure 4:**
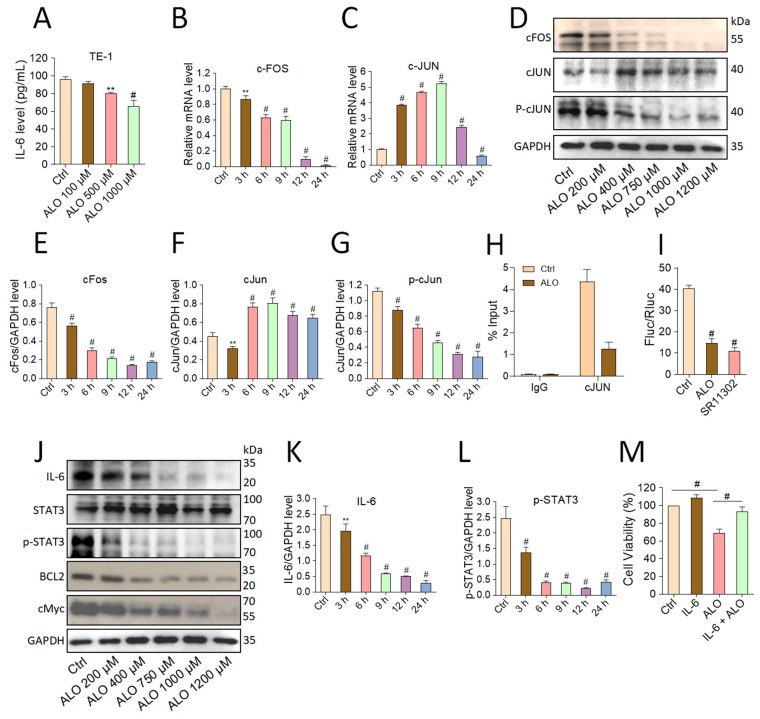
ALO Inhibits IL-6/JAK-STAT Signaling Pathway via Targeting AP-1. (**A**) ELISA analysis of IL-6 levels in the culture supernatant of TE-1 cells treated with ALO for 24 h. Data are presented as mean ± SD. (**B**,**C**) RT-qPCR analysis of *cFOS* and *cJUN* mRNA levels in TE-1 cells treated with ALO (600 μM) for the indicated times. Data are presented as the mean ± SD. (**D**) Western blot analysis of cFOS, cJUN, and phosphorylated cJUN (p-cJUN) in TE-1 cells treated with ALO for 24 h. GAPDH was used as a loading control. (**E**–**G**) Quantification of cFOS, cJUN and p-cJUN protein levels from (**D**), normalized to GAPDH. (**H**) Chromatin immunoprecipitation assay showing cJUN enrichment at the *IL-6* promoter in control and ALO-treated cells. Enrichment was quantified by qPCR and expressed as the percentage of input. (**I**) AP-1 luciferase reporter assay in TE-1 cells. Cells were co-transfected with an AP-1-responsive Firefly reporter and a Renilla internal control, then treated with DMSO, ALO (600 μM), or the specific AP-1 inhibitor SR11302 (2 μM) for 12 h. AP-1 activity was calculated as the Firefly/Renilla ratio. (**J**) Western blot analysis of IL-6, total STAT3, and phosphorylated STAT3 (p-STAT3), BCL2 and c-Myc in TE-1 cells treated with ALO for 24 h. GAPDH was used as a loading control. (**K**,**L**) Quantification of IL-6 and p-STAT3 protein levels normalized to GAPDH. (**M**) Viability changes of TE-1 cells treated with ALO (600 μM) in the presence or absence of exogenous IL-6 (20 ng/mL) for 12 h, measured by CCK-8. Data are presented as the mean ± SD. ** denotes *p* < 0.01, and # denotes *p* < 0.001 (one-way ANOVA with Tukey’s post-test). Original western blot images can be found in the Supplementary Materials.

To define how ALO suppresses IL-6 production, we first examined its effect on IL-6 protein levels in TE-1 cells. ELISA analysis showed that ALO reduced IL-6 secretion in a dose-dependent manner. Compared with the control group, treatment with 500 µM and 1000 µM ALO decreased IL-6 levels by approximately 30% and 45%, respectively (Figure 4A). Given that AP-1, composed mainly of cFOS and cJUN family proteins, is a key transcription factor regulating *IL-6*, we next investigated whether ALO modulates AP-1 activity. RT-qPCR analysis revealed distinct temporal dynamics. *cFOS* mRNA was consistently downregulated in a time-dependent manner (Figure 4B), whereas *cJUN* mRNA exhibited a biphasic response, transiently increasing at 6–9 h and then decreasing at 12–24 h (Figure 4C). Western blot analysis confirmed these results at the protein level. cFOS protein levels progressively decreased over time, whereas cJUN protein levels initially increased and then declined (Figure 4D–G). Importantly, phosphorylated cJUN (p-cJUN), the activated form of cJUN, was markedly suppressed at all examined time points, indicating that ALO impairs AP-1 transcriptional activity (Figure 4D,G).

To directly evaluate AP-1 binding to the *IL-6* promoter, we performed a chromatin immunoprecipitation assay. ALO treatment markedly reduced cJUN occupancy at the *IL-6* promoter (Figure 4H). Furthermore, AP-1 luciferase reporter assays demonstrated that ALO significantly reduced AP-1-driven luciferase activity, similar to the AP-1 inhibitor SR11302 (Figure 4I). These results suggest that ALO inhibits AP-1 binding to the IL-6 promoter and suppresses AP-1 transcriptional activity, thereby reducing IL-6 expression.

Because IL-6 activates downstream JAK-STAT signaling, we next assessed the effect of ALO on this pathway. Western blot analysis showed that IL-6 protein levels were significantly decreased after ALO treatment, consistent with the ELISA results. In parallel, phosphorylated STAT3 (p-STAT3) was markedly reduced, whereas total STAT3 levels remained unchanged (Figure 4J). Quantitative analysis confirmed that IL-6 and p-STAT3 levels declined in a time-dependent manner, with maximal inhibition observed at 24 h (Figure 4K,L). The protein levels of BCL2 and c-Myc, two downstream targets of STAT3, were also reduced (Figure 4J). Consistently, reductions in IL-6, p-STAT3 and p-cJUN were observed in ALO-treated KYSE-150 cells (Figure S5A).

To explore whether ALO affects upstream regulators of AP-1, we examined the phosphorylation of JNK and ERK, two major kinases involved in cJUN activation. ALO treatment progressively reduced the levels of phosphorylated JNK and ERK in TE-1 cells, while total JNK and ERK levels remained unchanged (Figure S5B). These results suggest that ALO suppresses AP-1 activity, at least in part, by inhibiting upstream JNK and ERK signaling.

Finally, we performed a rescue experiment to confirm whether IL-6 suppression contributes functionally to the anti-proliferative effect of ALO. Exogenous IL-6 significantly reversed the ALO-induced reduction in cell viability, indicating that IL-6 is a key downstream mediator of ALO activity (Figure 4M). These results suggest that ALO reduces IL-6 production and inhibits STAT3 signaling by targeting the AP-1 transcription factor.

### 3.5. ALO and Cisplatin Exert Synergistic Effects In Vitro and In Vivo

Cisplatin (CDDP) is a cornerstone of ESCC chemotherapy and primarily induces DNA-damage mediated apoptosis and ferroptosis. In contrast, our data indicate that ALO exerts its antitumor effect through a distinct mechanism involving suppression of the IL-6/STAT3 signaling axis. We therefore investigated whether combining these mechanistically distinct agents would produce synergistic antitumor activity.

**Figure 5:**
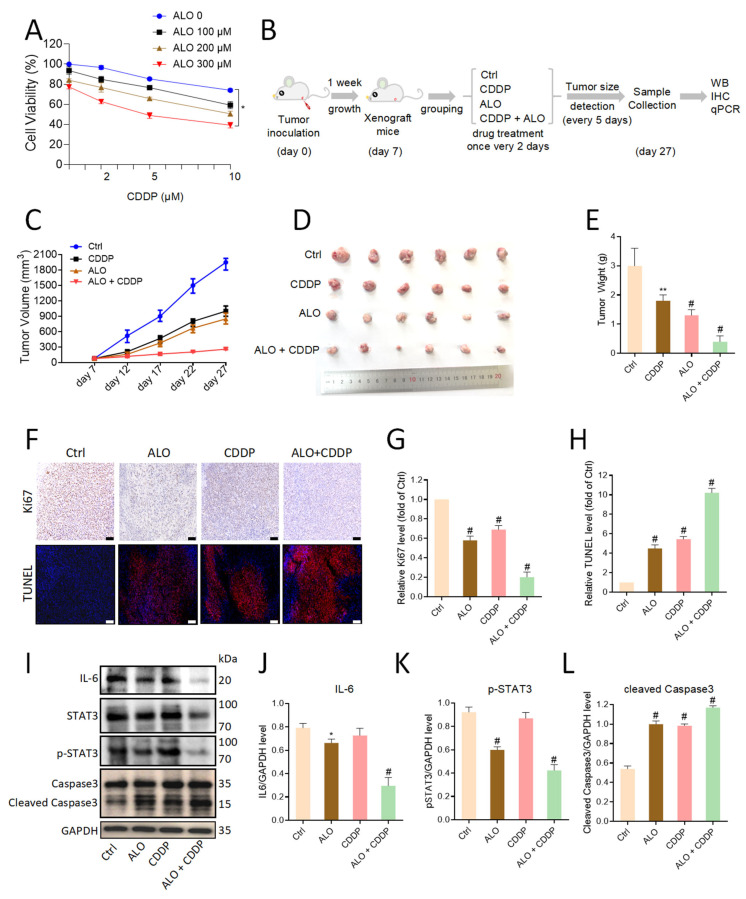
ALO and cisplatin exert synergistic antitumor effects in vitro and in vivo. (**A**) Effects of ALO and cisplatin (CDDP) on ESCC cell viability. AKR cells were treated with increasing concentrations of CDDP alone or in combination with ALO for 24 h, and cell viability was measured by CCK-8 assay. Data are presented as the mean ± SD, *n* = 3. (**B**) Schematic representation of the in vivo tumor model and treatment schedule. Mice were inoculated with AKR cells, allowed to grow for 7 days, randomized into four groups (Ctrl, CDDP, ALO, and ALO + CDDP) and treated as every 2 days for 20 days. Tumor size was measured every 5 days, and tissues were collected on day 27 for analysis. (**C**) Tumor growth curves. Data are presented as the mean tumor volume ± SD, *n* = 6 per group. (**D**) Representative images of excised tumors from each group at the endpoint. (**E**) Tumor weight quantification. Data are presented as the mean ± SD, *n* = 6 per group. (**F**) Representative Ki67 immunohistochemical staining and TUNEL staining of tumor sections. Scale bar is 100 μm. (**G**) Quantification of Ki67-positive cells. (**H**) Quantification of TUNEL-positive cells. Data are presented as the mean ± SD, *n* = 3. (**I**) Western blot analysis of IL-6, STAT3, phosphorylated STAT3, caspase-3, cleaved caspase-3, and GAPDH in tumor lysates. (**J**–**L**) Quantification of IL-6, phosphorylated STAT3, and cleaved caspase-3 protein levels normalized to GAPDH. Data are presented as the mean ± SD, *n* = 3. * denotes *p* < 0.05, ** denotes *p* < 0.01, and # denotes *p* < 0.001 (one-way ANOVA with Tukey’s post-test). Original western blot images can be found in the Supplementary Materials.

We first evaluated the combinatorial effects in vitro. Co-treatment with ALO significantly enhanced the cytotoxic effect of cisplatin in a dose-dependent manner, as measured by cell viability assay (Figure 5A). To quantitatively evaluate the synergistic effects of ALO and CDDP, the combination index (CI) was calculated using the Chou–Talalay method. As shown in Supplementary Figure S6A, the CI values for all tested concentration combinations were below 1, indicating strong synergistic effects.

Building on this in vitro synergy, we established a mouse tumor model to evaluate the antitumor efficacy of the combination in vivo. Tumor bearing mice were treated with vehicle control, ALO monotherapy, CDDP monotherapy, or ALO plus CDDP (Figure 5B). Longitudinal monitoring of tumor growth revealed that the combination treatment resulted in the most potent suppression of tumor growth throughout the treatment period, with final tumor volumes in the combination group being significantly smaller than those in either monotherapy group (Figure 5C). Representative tumor images at the endpoint visually confirmed the superior efficacy of the combination (Figure 5D), which was further quantified by the significantly lower tumor weight in the combination group compared with the other groups (Figure 5E). Importantly, mouse body weight and major organs were not significantly affected by the treatment, indicating an acceptable safety profile (Figure S6B,C).

To investigate the cellular mechanisms underlying the enhanced antitumor effect, we performed immunohistochemical analysis of tumor sections. Combination treatment markedly reduced the expression of the proliferation marker Ki67 and significantly increased the number of TUNEL-positive apoptotic cells, indicating that the synergistic effect involved both inhibition of proliferation and induction of apoptosis (Figure 5F–H).

At the molecular level, Western blot analysis of tumor lysates showed that combination therapy most effectively suppressed key pro-tumorigenic signaling pathways. Protein levels of IL-6 and phosphorylated STAT3 were most profoundly reduced in the combination group (Figure 5I–K). Furthermore, cleaved caspase-3 levels were highest in tumors from the combination group, consistent with the enhanced apoptotic response observed histologically (Figure 5I,L).

Taken together, these findings demonstrate that ALO synergizes with cisplatin to inhibit ESCC growth both in vitro and in vivo. This synergistic effect is associated with potent suppression of the IL-6/STAT3 survival pathway and robust induction of apoptosis.

## 4. Discussion

ESCC remains one of the most challenging malignancies worldwide, accounting for approximately 85% of esophageal cancer cases globally, with particularly high incidence in East Asia and Eastern Africa [28]. Despite advances in multimodal treatment strategies, the five-year survival rate for advanced ESCC remains poor, and the global burden is projected to reach 957,000 new cases annually by 2040 [28]. A major obstacle to effective treatment is the development of chemoresistance, which remains a critical clinical challenge [29,30]. The pressing need for novel therapeutic strategies has directed attention toward natural products with multitargeted bioactivities. In this study, we identified ALO, a quinolizidine alkaloid derived from *Sophora alopecuroides* L., as a potent antitumor agent that suppresses ESCC progression. Our findings demonstrate that ALO exerts its anti-cancer effects through disruption of the oncogenic IL-6/STAT3 signaling axis by targeting the upstream AP-1 transcription factor complex and induction of apoptosis in ESCC cells. Furthermore, we provide solid evidence that ALO synergizes with cisplatin, a standard-of-care chemotherapeutic for ESCC, both in vitro and in vivo, highlighting its potential as a chemosensitizing agent for ESCC treatment.

Our initial phenotypic screening revealed that ALO selectively inhibits the viability and clonogenicity of ESCC cells in a dose- and time-dependent manner while exerting only marginal inhibitory effects on normal human esophageal epithelial cells. This selectivity is consistent with previous reports in other cancer types, such as non-small-cell lung cancer and colorectal cancer [20,22], where ALO exhibited preferential cytotoxicity toward malignant cells. Mechanistically, we established that ALO triggers the intrinsic apoptotic pathway, as evidenced by loss of mitochondrial membrane potential, cleavage of caspase-3, and cleavage of PARP1. This apoptotic response was effectively attenuated by the pan-caspase inhibitor Q-VD-Oph, confirming that ALO-induced apoptosis is at least partly caspase-dependent. These findings are consistent with studies in hepatocellular carcinoma [23], osteosarcoma [31], and prostate cancer [24], in which ALO similarly activated mitochondrial apoptosis. In addition, ALO has been shown to induce apoptosis in multiple myeloma through dual activation of caspase-8 and caspase-9 pathways [32], further supporting the broad pro-apoptotic activity of this compound.

To elucidate the molecular basis of the anti-ESCC activity of ALO, we performed unbiased transcriptomic analysis. KEGG enrichment analysis of downregulated genes revealed a striking suppression of inflammation-related pathways, most notably the cytokine–cytokine receptor interaction and JAK-STAT signaling pathways. Among the inflammatory mediators identified, IL-6 was the most consistently downregulated target at both the mRNA and protein levels. This finding is particularly relevant given the well-documented role of IL-6 as a master regulator of ESCC progression, promoting tumor cell proliferation, survival, invasion and chemoresistance through autocrine and paracrine mechanisms [27,33]. Recent studies have further shown that the ESCC tumor microenvironment is characterized by complex interactions between cancer cells and stromal components, including cancer-associated fibroblasts (CAFs), which secrete IL-6 and other inflammatory cytokines [34]. Moreover, Chen et al. demonstrated that ALO suppresses the IL-6/JAK1/STAT3 feedback loop in cervical cancer [35], providing independent support for our mechanistic findings and suggesting that inhibition of the IL-6/STAT3 axis may represent a conserved anti-cancer mechanism of ALO across multiple tumor types.

While ALO has been previously reported to exert anti-inflammatory effects via NF-κB inhibition in macrophages [25], models of colitis [36], and allergic airway inflammation [37], our data suggest a distinct mechanism in ESCC. We observed that ALO did not significantly alter the expression of several canonical NF-κB-related targets, including *NFKBIA*, *CXCL1*, and *TNFRSF11B*. Instead, ALO suppressed the AP-1 transcription factor complex, as reflected by reduced cFOS expression, impaired cJUN phosphorylation, decreased binding of cJUN to the IL-6 promoter region, and diminished AP-1 transcriptional activity. This AP-1-centered mechanism has not been extensively characterized for ALO in cancer, although AP-1 modulation has been implicated in the protective effects of ALO against renal injury [38]. Our findings suggest that AP-1 functions as an important upstream node through which ALO reduces IL-6 transcription, representing a mechanistic divergence from its previously described NF-κB-associated anti-inflammatory effects.

The downstream consequences of IL-6 suppression were substantial. ALO treatment led to a marked reduction in STAT3 phosphorylation, a key signaling event downstream of IL-6/JAK activation. The IL-6/STAT3 axis is a central oncogenic driver in ESCC and is closely associated with poor prognosis, tumor progression, and therapeutic resistance [27]. The functional relevance of this axis was further supported by rescue experiments, in which exogenous IL-6 significantly reversed the anti-proliferative effects of ALO. This finding indicates that IL-6/STAT3 suppression is an important contributor to the anti-ESCC activity of ALO. Notably, ALO did not markedly affect total STAT3 levels but specifically inhibited STAT3 activation, supporting pathway-specific regulation rather than general suppression of STAT3 expression. Consistent with our findings, ALO has been shown to inhibit STAT3 phosphorylation in colorectal cancer through regulation of the circNSUN2/miR-296-5p/STAT3 pathway [21,39], as well as in psoriasis models, in which ALO suppressed Th17 differentiation by inhibiting STAT3 phosphorylation [40].

A critical and clinically relevant finding of our study is the synergistic interaction between ALO and cisplatin. Cisplatin-based regimens remain a cornerstone of ESCC chemotherapy; however, their efficacy is frequently limited by intrinsic or acquired resistance [14]. The mechanisms underlying cisplatin resistance in ESCC are multifaceted and include activation of pro-survival pathways, enhanced DNA repair, metabolic reprogramming, and evasion of apoptosis [41,42,43]. Landmark clinical trials have established that the addition of immune checkpoint inhibitors to chemotherapy significantly improves outcomes in ESCC. For example, the CheckMate 648 trial demonstrated that nivolumab plus chemotherapy significantly improved overall survival in patients with advanced ESCC [44], while the ESCORT-NEO trial showed that neoadjuvant camrelizumab plus chemotherapy achieved superior pathological complete response rates compared to chemotherapy alone [45]. These paradigm-shifting studies highlight the importance of targeting immune and inflammatory pathways in ESCC treatment.

Our data demonstrate that combining ALO with cisplatin resulted in superior tumor growth inhibition compared to either monotherapy in vivo. This enhanced anti-tumor effect was associated with stronger suppression of the IL-6/STAT3 axis and more robust induction of apoptosis, as evidenced by increased cleaved caspase-3 expression and TUNEL-positive cells, together with decreased Ki67 expression. This chemosensitizing effect is consistent with recent observations in other cancer models. For instance, ALO has been reported to reverse cisplatin resistance in colorectal cancer [46], and a related quinolizidine alkaloid derivative has been explored for enhancing oxaliplatin sensitivity [47]. Together, these findings suggest that ALO may improve the therapeutic efficacy of platinum-based chemotherapy by attenuating survival signaling and promoting apoptotic responses.

The safety profile of ALO is an important consideration for its potential translational development. In our in vivo studies, combination treatment did not cause significant body weight loss or overt histopathological abnormalities in major organs, suggesting acceptable tolerability under the experimental conditions used. This observation is consistent with the favorable safety profile reported in other studies [32,48]. Notably, Qiu et al. demonstrated that aloperine protects against cisplatin‑induced kidney injury, suggesting that aloperine may have a favorable safety profile and could even alleviate nephrotoxicity when combined with cisplatin [49]. Nevertheless, further pharmacokinetic, pharmacodynamic, and toxicological studies are required before clinical translation can be considered. In particular, given the relatively high concentrations of ALO required for in vitro efficacy, future work should evaluate its bioavailability, achievable plasma and tumor concentrations, formulation optimization, and therapeutic window in clinically relevant models.

Although our study supports the anti-ESCC activity of ALO, several limitations should be noted. First, the direct molecular target of ALO remains unknown. Although our data implicate AP-1 and upstream MAPK signaling in ALO-mediated IL-6 suppression, whether ALO directly binds AP-1 components, upstream kinases, or other regulatory proteins requires further investigation. Second, RNA-seq analysis indicated that ALO also modulates other pathways, including TNF and MAPK signaling, which may contribute to its anti-tumor effects. Third, although ALO synergized with cisplatin in vitro and in vivo, the optimal dosing schedule, administration sequence, and long-term efficacy remain to be defined in more clinically relevant models, such as patient-derived xenografts, organoids, and immune-competent ESCC models [50,51]. Finally, the role of AP-1 should be further validated through genetic perturbation, and future studies should determine whether ALO affects IL-6 production by stromal or immune cells in the ESCC tumor microenvironment.

## 5. Conclusions

In conclusion, this study establishes ALO as a promising therapeutic agent for ESCC. We have elucidated a previously unrecognized mechanism of action wherein ALO targets the AP-1 transcription factor complex, leading to the suppression of IL-6 production and subsequent inhibition of the oncogenic JAK/STAT3 signaling pathway. This pathway-specific inhibition results in potent anti-proliferative and pro-apoptotic effects against ESCC cells. Most importantly, we demonstrate that ALO synergizes with cisplatin, significantly enhancing its antitumor efficacy both in vitro and in vivo, while exhibiting a favorable safety profile. These findings not only highlight the therapeutic potential of ALO as a monotherapy for ESCC but also provide a compelling rationale for its use as a chemosensitizing agent to overcome cisplatin resistance. Given the urgent clinical need for more effective treatment strategies for advanced ESCC, particularly in light of the recent success of immunotherapy combinations and the persistent challenge of chemoresistance, our study positions ALO as a promising lead compound worthy of further preclinical and clinical investigation, either alone or in combination with existing chemotherapeutic and immunotherapeutic regimens.

## Figures and Tables

**Table 1:** Primers used for quantitative real-time PCR analysis.

Gene Name	Primer Sequence (5′ → 3′)	Product (bp)
*GAPDH*	F: TCTATAAATTGAGCCCGCAGCC R: ACCAAATCCGTTGACTCCGA	22 20
*IL6*	F: CCACCGGGAACGAAAGAGAA R: GAGAAGGCAACTGGACCGAA	20 20
*IL11*	F: GCGGACAGGGAAGGGTTAAAG R: AGGCGGCAAACACAGTTCAT	21 20
*CXCL1*	F: TTTCTGAGGAGCCTGCAACA R: GCACATACATTCCCCTGCCT	20 20
*NFKBIA*	F: GAAGTGATCCGCCAGGTGAA R: CTCACAGGCAAGGTGTAGGG	21 21
*TNFRSF11B*	F: ACAGCAAAGTGGAAGACCGT R: CCTTCCTTGCATTCGCACAC	20 20
*cFOS*	F: TGGCGTTGTGAAGACCATGA R: AGTTGGTCTGTCTCCGCTTG	20 21
*cJUN*	F: GGAGGGAGGTTTGTGAGAGC R: ACAAACAACACTGGGCAGGA	20 20

Note: F, forward primer; R, reverse primer; bp, base pairs.

## Data Availability

The raw RNA-seq data generated in this study have been deposited in the Genome Sequence Archive (GSA) at the National Genomics Data Center (NGDC), China National Center for Bioinformation (CNCB), under accession number PRJCA061061. The data will be publicly released upon publication of this manuscript.

## Supplementary Materials

The following supporting information can be downloaded at: https://www.mdpi.com/article/10.3390/biom16060791/s1, Figure S1. Related to Figure 1. Figure S2. Related to Figure 2. Figure S3. Related to Figure 3. Figure S4. IL6 expression in ESCC cell lines. Figure S5. Related to Figure 4. Figure S6. Related to Figure 5. File S1. Original image of Western Blot.

## Abbreviations

The following abbreviations are used in this manuscript:

ALO	Aloperine
AP-1	Activator protein-1
CCK-8	Cell Counting Kit-8
CDDP	Cisplatin
c-FOS	FBJ murine osteosarcoma viral oncogene homolog
c-JUN	Jun proto-oncogene
GAPDH	Glyceraldehyde-3-phosphate dehydrogenase
IL-6	Interleukin-6
KEGG	Kyoto Encyclopedia of Genes and Genomes
NF-κB	Nuclear factor kappa-light-chain-enhancer of activated B cells
PARP1	Poly(ADP-ribose) polymerase 1
RT-qPCR	Quantitative real-time PCR
STAT3	Signal transducer and activator of transcription 3
TNF	Tumor necrosis factor
TUNEL	Terminal deoxynucleotidyl transferase dUTP nick end labeling
ΔΨm	Mitochondrial membrane potential
