# Supplementary material for: Aloperine Suppresses the Tumorigenicity of Esophageal Squamous Cell Carcinoma by Targeting the AP-1/IL-6/STAT3 Signaling Axis
Source: Biomolecules. 2026 May 27;16(6):791. doi: 10.3390/biom16060791 (PMC13297383; doi:10.3390/biom16060791)
Supplement: Supplementary file 1 [file biomolecules-16-00791-s001.zip › biomolecules-4272509-supplementary.pdf]

Supplementary Information for

Aloperine Suppresses the Progression of Esophageal Squamous Cell Carcinoma by Targeting the IL-6/STAT3 Signaling Axis

Ba-Fang Ma[1,2], Jun-Nan Ye[1,2], Die Bai[1,2], Chang Ge[1,2], Yingchao Guan[2,3], Yang Lou[1,2], Ya-Ping Liang[2,3], Na Bu[4], Wenhui Hao[2,3], Yasen Maimaitiyiming[1,2,5]*

[1]Department of Immunology, School of Basic Medical Sciences, Xinjiang Medical University, Urumqi, 830011, China;

[2]Xinjiang Key Laboratory of Molecular Biology for Endemic Diseases, Xinjiang Medical University, Urumqi, 830011, China;

[3] Department of Biochemistry and Molecular Biology, School of Basic Medical Sciences, Xinjiang Medical University, Urumqi, Xinjiang, China;

[4]Department of Pharmacy, Women's Hospital, Zhejiang University School of Medicine, Hangzhou, China;

[5]Institute of Basic Medical Sciences, School of Basic Medical Sciences, Xinjiang Medical University, Urumqi, 830011, China.

*Correspondence: Yasen Maimaitiyiming (yasinjan@zju.edu.cn; ysjm@xjmu.edu.cn).

This pdf file includes 6 Supplementary Figures and Figure legends.

Figure S1

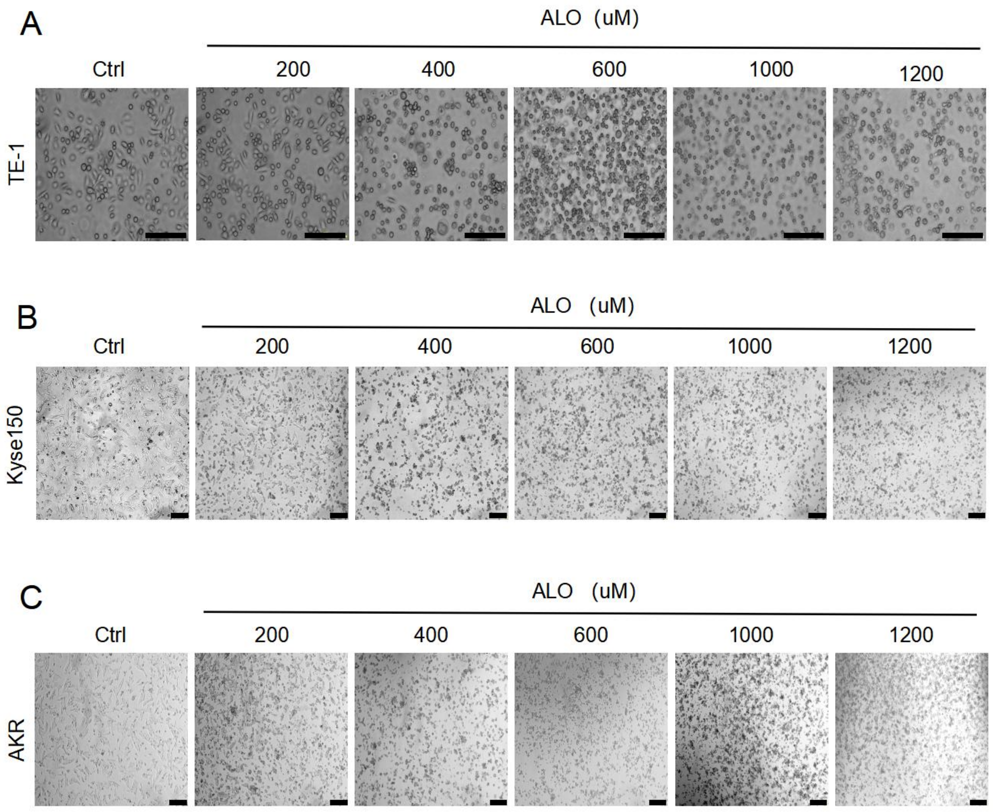

Figure S1. Related to Figure 1. (A-C) Light microscopy images of TE-1 (A), KYSE-150 (B), and AKR (C) cells treated with 0-1200 μM ALO for 24 h. Scale bar is 200 μm.

Figure S2

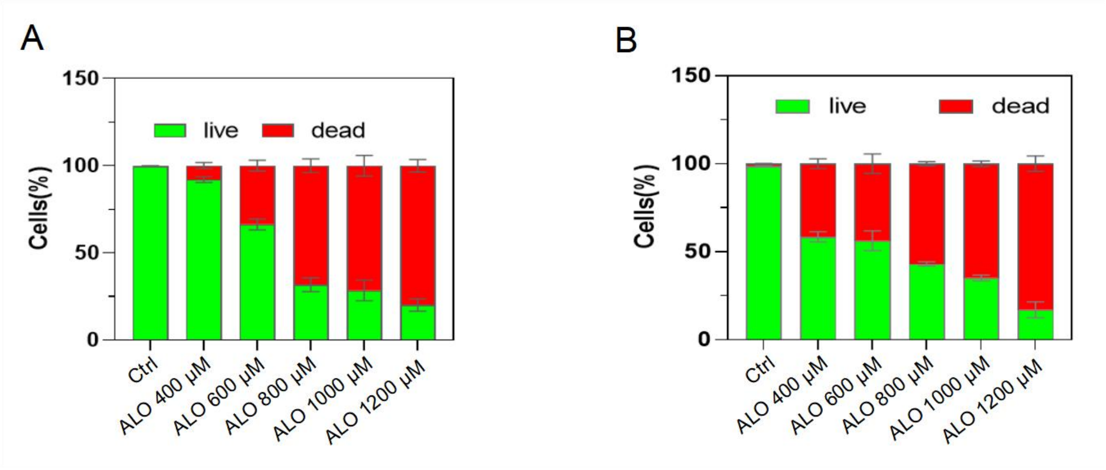

Figure S2. Related to Figure 2. Quantification of live/dead staining from Figure 2 A and B.

Figure S3

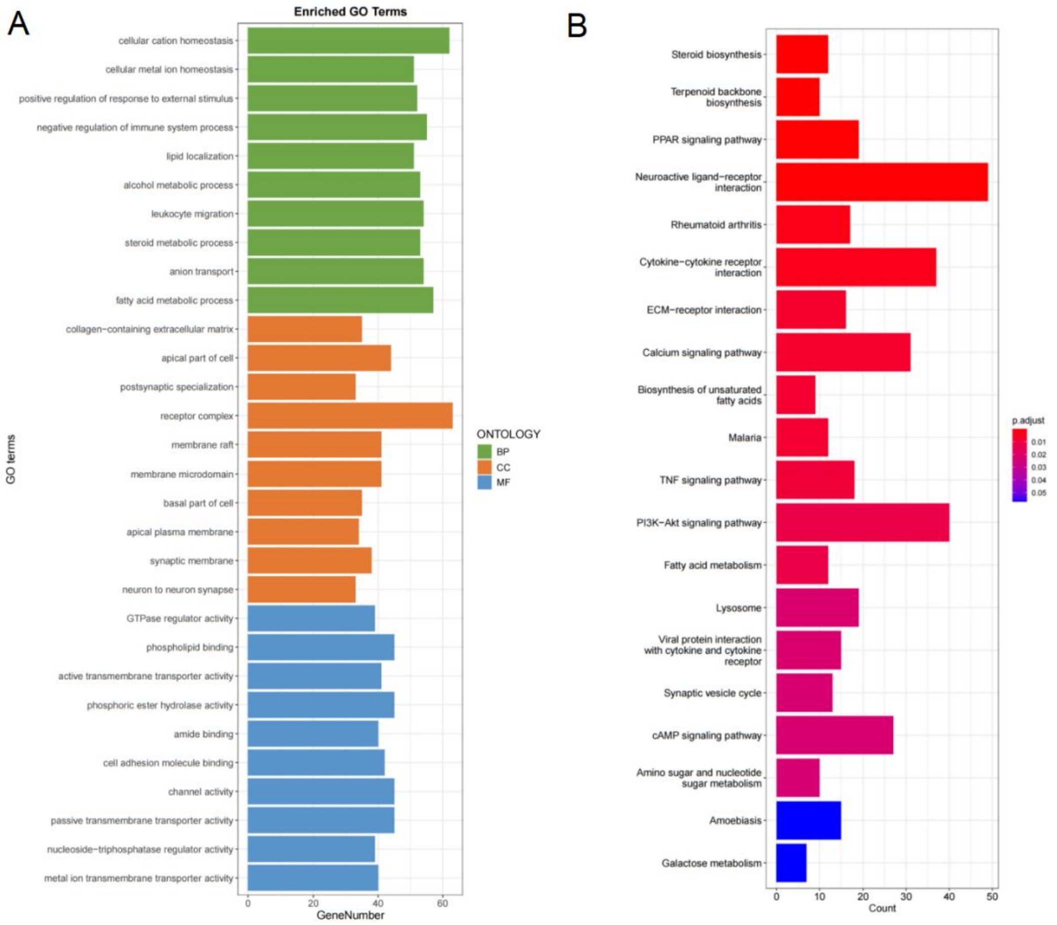

Figure S3. Related to Figure 3 . (A) GO enrichment analysis of all DEGs. Biological Process (BP) terms are shown in green, Cellular Component (CC) in orange, and Molecular Function (MF) in blue. Bar length represents gene count; color represents adjusted p-value. (B) KEGG pathway enrichment analysis of all DEGs. Bar length indicates gene count; color indicates adjusted p-value.

Figure S4

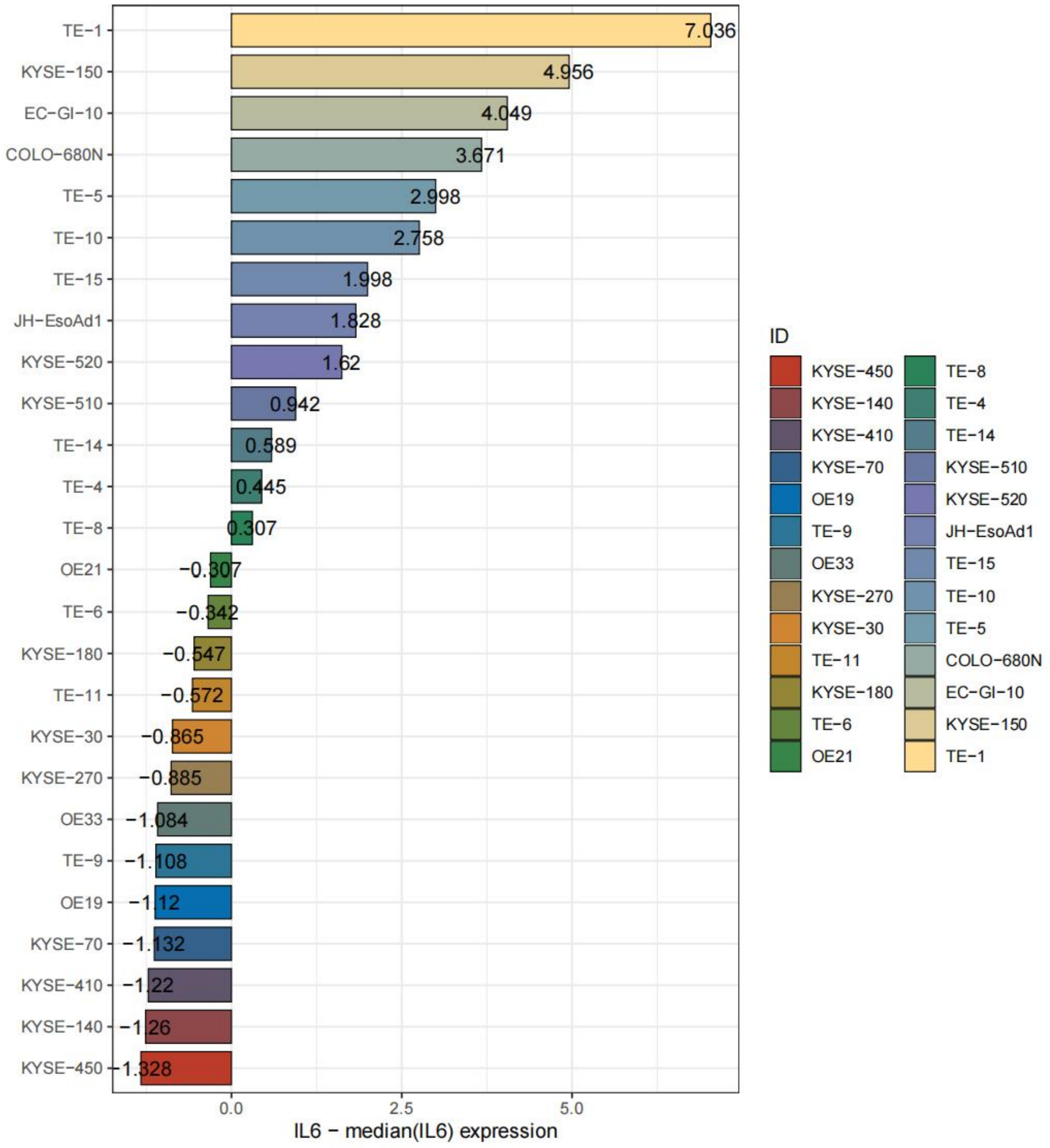

Figure S4. IL6 expression in ESCC cell lines. Circular bar chart showing the distribution of gene expression across various tumor cell lines. Each bar represents an individual cell line, and the bar height indicates the relative gene expression level. Expression data of ESCC cell lines were obtained from the Cancer Cell Line Encyclopedia (CCLE) dataset (https://depmap.org/portal/data_page/?tab=allData). Statistical analyses were performed using R software (version 4.0.3).

Figure S5

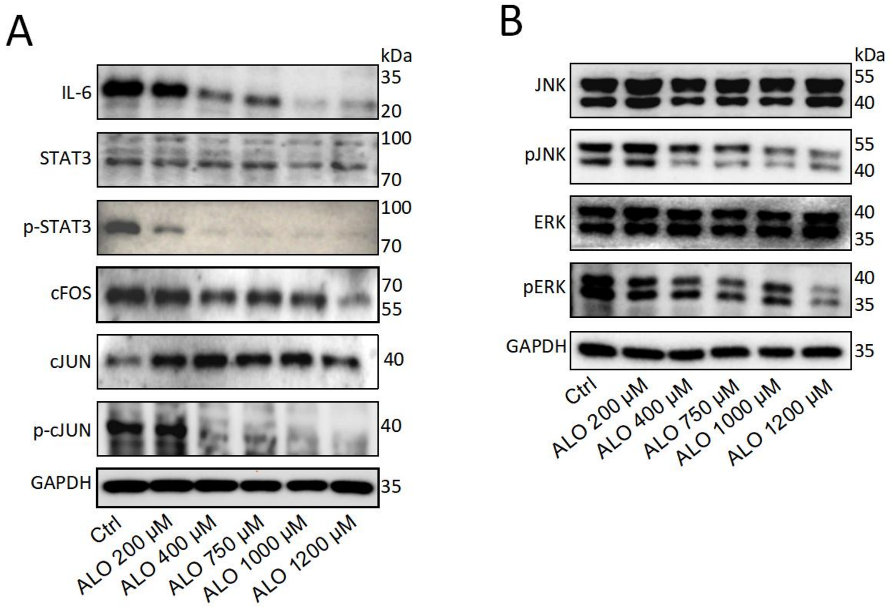

Figure S5. Related to Figure 4. (A) Western blot analysis of IL-6, p-STAT3, total STAT3, p-cJUN, c-JUN, and c-FOS in KYSE150 cells treated with ALO for 24 h. (B) Western blot analysis of JNK, pJNK, ERK, and pERK in TE-1 cells treated with ALO for 24 h. GAPDH was used as a loading control.

Figure S6

A

ALO (μM)	CDDP (μM)	CI
100.0	2.0	0.81892
100.0	5.0	0.87635
100.0	10.0	0.7746
200.0	2.0	0.89055
200.0	5.0	0.81515
200.0	10.0	0.74289
300.0	2.0	0.74887
300.0	5.0	0.62153
300.0	10.0	0.6309

B

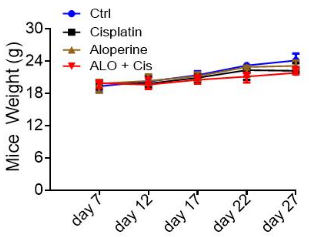

C

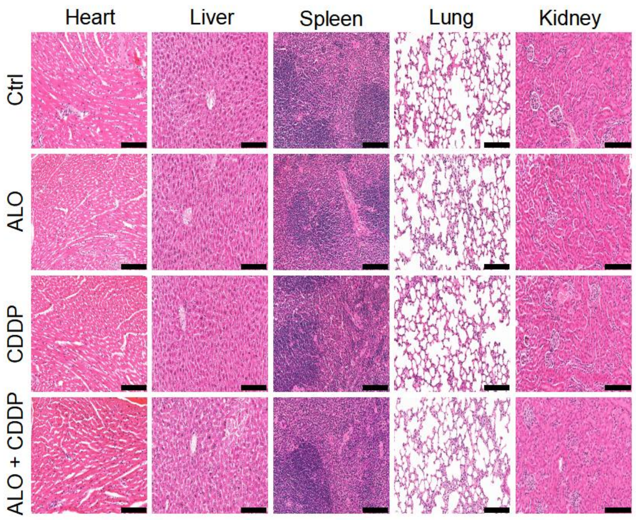

Figure S6. Related to Figure 5. (A) Combination index (CI) analysis of the synergistic effect between ALO and cisplatin (CDDP). AKR cells were treated with increasing concentrations of ALO and CDDP alone or in combination for 24 h, and cell viability was measured by CCK-8 assay (as shown in Figure 5A). The CI values were calculated using CompuSyn software based on the Chou-Talalay method. (B) Body weight changes of mice over time. Mice were weighed every 5 days during treatment. Mean body weight ± SD (n = 6 per group); no significant differences observed among groups ($P > 0.05$). (C) Histological analysis of major organs: Hematoxylin and eosin (H&E)-stained sections of heart, liver, spleen, lung, and kidney from each treatment group. Scale bar is 20 μm.
