# Supplementary figures and images for: Aloperine Suppresses the Tumorigenicity of Esophageal Squamous Cell Carcinoma by Targeting the AP-1/IL-6/STAT3 Signaling Axis
Source: Biomolecules. 2026 May 27;16(6):791. doi: 10.3390/biom16060791 (PMC13297383; doi:10.3390/biom16060791)

Figure 2K

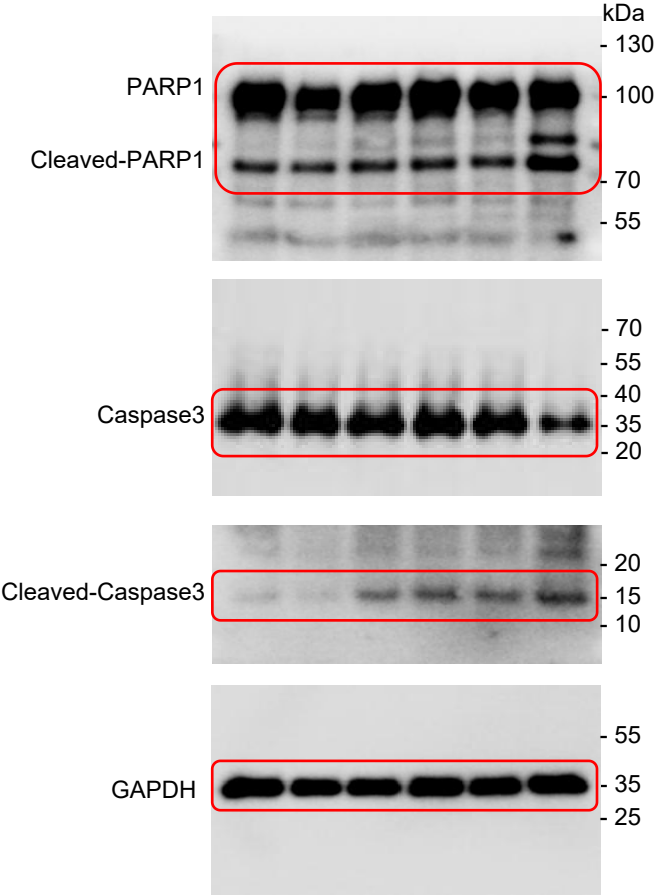

Figure 4D

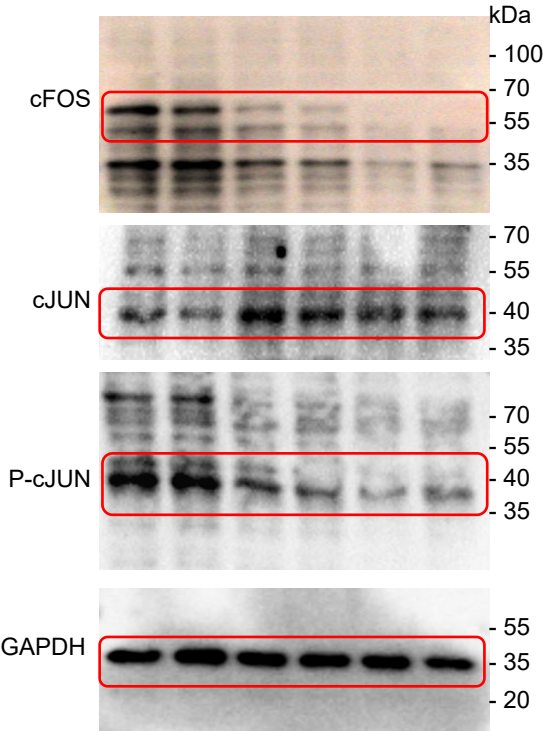

Figure 4J

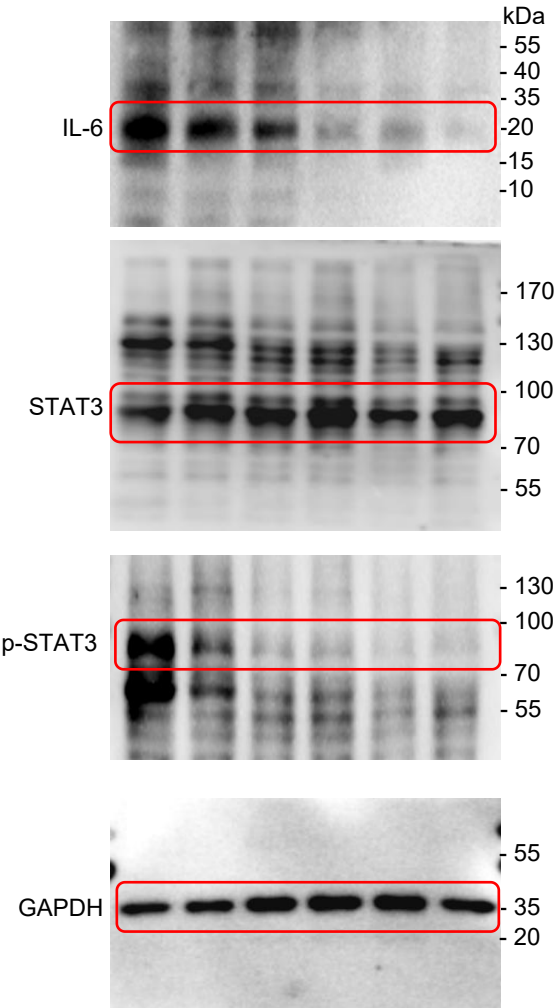

Figure S5

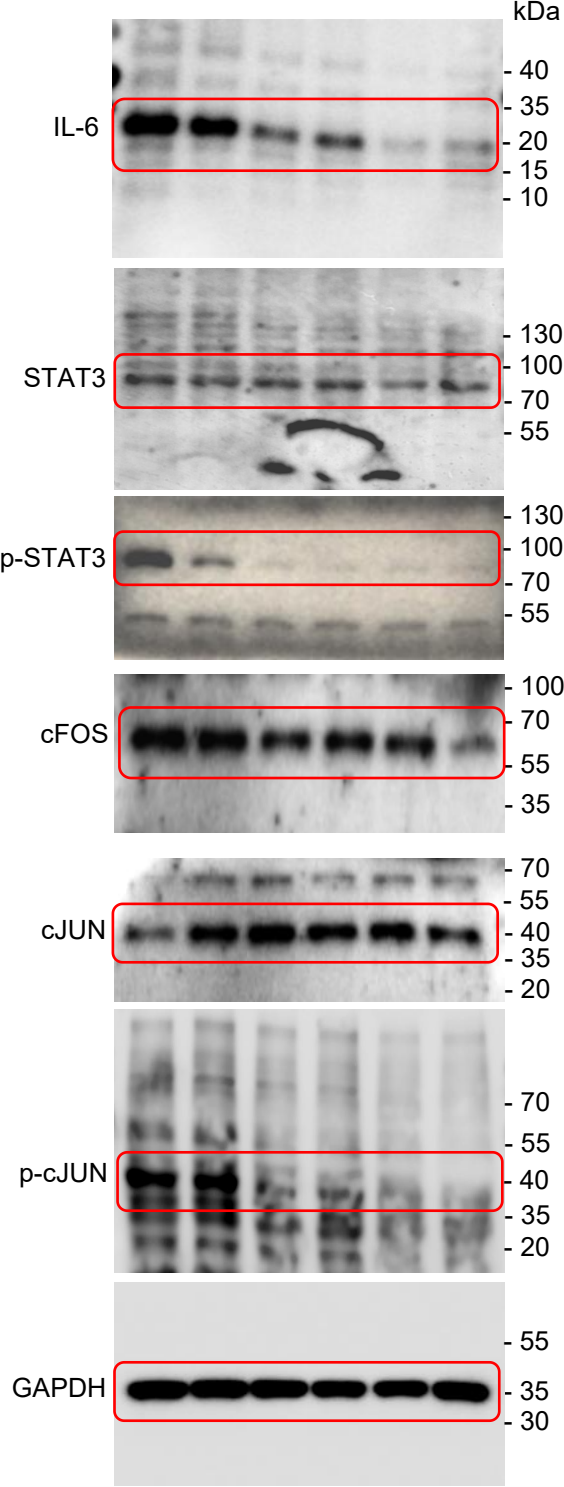

Figure 5l

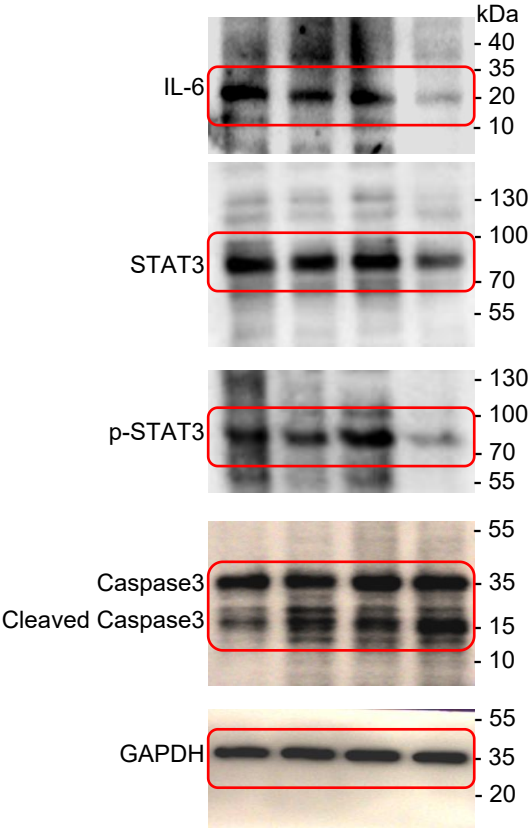

Supplement: Supplementary file 1 [file biomolecules-16-00791-s001.zip › biomolecules-4272509-original-images.pdf]
